# Development of Advanced Chemometric-Assisted Spectrophotometric Methods for the Determination of Cromolyn Sodium and Its Alkaline Degradation Products

**DOI:** 10.3390/molecules25245953

**Published:** 2020-12-16

**Authors:** Noha M. El Zahar, Mariam M. Tadros, Bassam M. Ayoub

**Affiliations:** 1 Pharmaceutical Analytical Chemistry Department, Faculty of Pharmacy, Ain Shams University, Organization of African Unity Street, Abassia, Cairo 11566, Egypt; mariam.tadros@pharma.asu.edu.eg; 2 Medicinal Chemistry Department, Faculty of Pharmacy, King Salman International University, Ras-Sedr, South Sinai Governorate 46612, Egypt; 3 Pharmaceutical Chemistry Department, Faculty of Pharmacy, The British University in Egypt, El-Sherouk City, Cairo 11837, Egypt; bassam.ayoub@bue.edu.eg; 4 The Center for Drug Research and Development (CDRD), Faculty of Pharmacy, The British University in Egypt, El-Sherouk City, Cairo 11837, Egypt

**Keywords:** cromolyn sodium, chemometrics, mean centering ratio spectra, multivariate calibration, stability indicating

## Abstract

Advanced and sensitive spectrophotometric and chemometric analytical methods were successfully established for the stability-indicating assay of cromolyn sodium (CS) and its alkaline degradation products (Deg1 and Deg2). Spectrophotometric mean centering ratio spectra method (MCR) and chemometric methods, including principal component regression (PCR) and partial least square (PLS-2) methods, were applied. Peak amplitudes after MCR at 367.8 nm, 373.8 nm and 310.6 nm were used within linear concentration ranges of 2–40 µg mL^−1^, 5–40 µg mL^−1^ and 10–100 µg mL^−1^ for CS, Deg1 and Deg2, respectively. For PCR and PLS-2 models, a calibration set of eighteen mixtures and a validation set of seven mixtures were built for the simultaneous determination of CS, Deg1 and Deg2 in the ranges of 5–13 µg mL^−1^, 8–16 µg mL^−1^, and 10–30 µg mL^−1^, respectively. The authors emphasize the importance of a stability-indicating strategy for the investigation of pharmaceutical products.

## 1. Introduction

**Figure 1:**
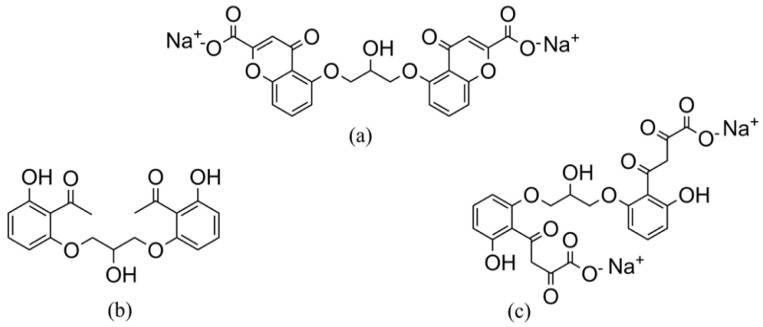
Chemical structures of (**a**) CS, (**b**) Deg1 and (**c**) Deg2.

Cromolyn sodium (CS), 5,5’-[(2-hydroxytrimethylene)dioxy]bis[4-oxo-4H-1-benzopyran-2-carboxylate], is a chromone derivative possessing two carboxylic groups (Figure 1a). It is considered to be a fascinating antiallergic agent for treating asthma and allergic rhinitis as it inhibits the release of inflammatory chemicals [1,2,3]. Moreover, CS is responsible for neuro-protection in the mouse model of amyotrophic lateral sclerosis by decreasing the inflammatory response and delaying the neurological symptoms. CS inhibits mast cell degranulation and decreases renal cyst disease. It also has other effects on specific organs, including the liver [1,2,3].

The main degradation pathway of CS was found to be alkaline hydrolysis in aqueous and alcoholic solution [4,5]. CS alkaline degradation in aqueous solution yields Deg1, (1-{2-[3-(2-Acetyl-3-hydroxy-phenoxy)-2-hydroxypropoxy]-6-hydroxy-phenyl}-ethanone), as a reported pharmacopeial impurity [6] (Figure 1b). The degradation pathway of Deg1 follows a similar degradation pathway to khellin [7,8,9]. CS alkaline degradation in alcoholic solution yields Deg2, 4-(2-{3-[2-(3-Carboxy-3-oxo-propionyl)-3-hydroxy-phenoxy]-2-hydroxy-propoxy}-6-hydroxy-phenyl)-2,4-dioxo-butyric acid tetra-sodium salt (Figure 1c) [9,10]. This degradation involves opening of the γ-pyrone ring of the chromone nucleus and finally the formation of a bisacetophenone derivative [4,5]. Hence, it is useful and practical to assess CS in the presence of its chief degradation products, either Deg1 or Deg2, which would be of great importance in the routine analysis of CS for pharmaceutical quality control as well as clinical studies.

Determination of CS alone or in combination with other drugs such as fluorometholone and oxymetazoline was available in the literature, including spectroscopy [11,12,13,14,15,16], electrochemical methods [17,18,19,20], capillary electrophoresis [21], thin layer chromatography (TLC) [13,22,23] and HPLC using UV [23,24,25,26,27,28], fluorescence [29] and tandem mass [30,31,32] detectors. Spectroscopy provides major advantages that include simplicity and the lack of need to prepare the stationary and mobile phases, which are time consuming and costly, over all the mentioned chromatographic methods. Moreover, the development of new simple spectrophotometric and chemometric methods is necessary to give the researchers a set of “varieties” that they can use according to the laboratory limitations, especially in developing countries, in addition to being a greener method due to the lower consumption of toxic solvents and lower waste production. The literature review reveals that spectrophotometric derivative ratio spectra [4] and HPLC with UV detector [5,33,34,35,36,37] methods were used for the analysis of CS with its degradates and related substances. The purpose of the current investigation is to provide a new CS and its degradates (Deg1 and Deg2) method with fully detailed validation according to ICH guidelines as an alternative for the high-cost methods for the analysis of CS.

In developing countries, spectrophotometry continues to be popular due to its simplicity, the common availability of the instrumentation, the low cost and the fact that the procedures are not time and labor consuming [38,39,40,41,42]. Hence, in this work, advanced spectrophotometric mean centering ratio spectra method (MCR) and chemometric techniques were suggested for the assessment of CS and its alkaline degradates to determine this mixture in a simple and efficient way. For the chemometric methods, spectral analysis including many spectral wavelengths instead of using a single wavelength was applied, thus improving the precision, accuracy and selectivity. Two different multivariate calibration methods (PCR and PLS-2) were applied in this respect.

## 2. Methods

### 2.1. Instrumentation, Chemicals and Reagents

A Shimadzu spectrophotometer (1601, Kyoto/Japan) and a Jenway pH meter (Jenway Instruments Ltd, Essex, UK) were used. Regarding the calculation handling, Minitab (14.12.0) and MATLAB (7.0.1.24704, R14) were used. CS (99.61%) and Epicrom (40 mg/mL, batch 1203205) were provided by EIPICO (Egypt). Potassium hydroxide, sodium hydroxide and glacial acetic acid were obtained from SIGMA (Egypt, SAE).

### 2.2. Preparation of the CS Alkaline Degradation Products

CS alkaline degradate (Deg1) was prepared by refluxing 500 mg of CS for two hours with ten mL of 10% aqueous KOH followed by acidification using glacial acetic acid. Then the precipitate was filtered, washed and dried. While Deg2 was prepared by heating of 500 mg of CS for one hour at 70 °C with ten mL 10% alcoholic NaOH followed by acidification using glacial acetic acid to pH = 7.5. An orange-yellow solid was precipitated, filtered and dried [5,7,10]. All degradation products were confirmed based on TLC and UV spectrum analysis.

### 2.3. Procedures

**Figure 2:**
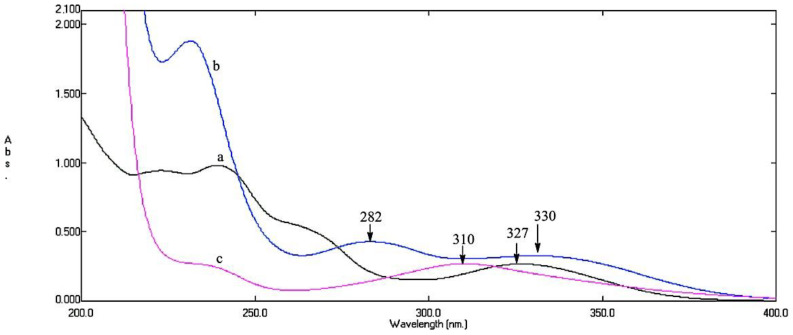
Zero-order absorption spectra of (**a**) 20 μg mL^−1^ CS, (**b**) 30 μg mL^−1^ Deg1 and (**c**) 30 μg mL^−1^ Deg2.

As a preliminary investigation, aliquots of working solutions of CS (20 µg mL^−1^), Deg1 (30 µg mL^−1^) and Deg2 (30 µg mL^−1^) were recorded over the range 200–400 nm, using water as a blank (Figure 2) showing their maximum wavelengths.

#### 2.3.1. Procedure for MCR Method

The scanned zero-order spectra of 2–40 µg mL^−1^, 5–40 µg mL^−1^ and 10–100 µg mL^−1^ for CS, Deg1 and Deg2, respectively were divided by a standard spectrum of 30 μg mL^−1^ of Deg1 to obtain the first ratio spectra of CS and Deg2 which were then mean centered, and also by a standard spectrum of 30.00 μg mL^−1^ of CS to obtain the first ratio spectra of Deg1 which were then mean centered. These mean centered ratio spectra of CS, Deg1, and Deg2, were subsequently divided by the mean centered ratio of αDeg2/αDeg1, αDeg2/αCS and αCS/αDeg1 analogous to 30 μg mL^−1^ each, respectively.

Eventually, the mean centered values of the second ratio spectra at 367.8, 373.8, and 310.6 nm (peak to peak) for CS, Deg1 and Deg2, respectively, were measured and plotted against the corresponding concentrations to construct their respective calibration curves.

#### 2.3.2. Procedure for PCR and PLS-2

A multilevel multifactorial calibration set of eighteen mixtures and a validation set of seven mixtures were built up for the simultaneous determination of CS, Deg1 and Deg2 in the ranges of 5–13 µg mL^−1^, 8–16 µg mL^−1^, and 10–30 µg mL^−1^, respectively, after the samples’ recording in the range 230–400 nm at 0.2 nm interval. The recorded spectra were then transferred to MATLAB for subsequent data analysis, using the PLS Toolbox and the calibration models (PCR and PLS-2) were constructed.

#### 2.3.3. Assay of Pharmaceutical Formulation (Epicrom Eye Drop)

A sample of 0.5 mL was accurately transferred from Epicrom eye drop to a 100 mL volumetric flask and diluted to the mark with distilled water to get 200.00 μg mL^−1^ of CS followed by serial dilution to the required concentrations. Standard addition technique was applied and known amounts of CS were added to the corresponding prepared solution of the pharmaceutical formulation. The absorbance spectra were recorded. MCR, PCR and PLS-2 procedures were adopted as mentioned under Section 2.3.1 and Section 2.3.2, and the percentage recoveries and standard deviation were calculated.

## 3. Results and Discussion

Zero-order absorption spectra of 20 μg mL^−1^ CS, 30 μg mL^−1^ Deg1 and 30 μg mL^−1^ Deg2 are illustrated in (Figure 2). Deg1 spectra showed the presence of two absorption bands at 282.0 and 330.0 nm instead of the characteristic absorption maximum of CS at 327.0 nm while Deg2 spectra showed a hypsochromic shift to 310.0 nm [4,5]. The Figure displays severely overlapped spectra which could not be clearly resolved. The adopted spectrophotometric and chemometric methods have the advantage of being simple, accurate and precise for the determination of CS with its alkaline degradates (Deg1 and Deg2) without any interference.

### 3.1. MCR Method

The MCR method compared to other spectrophotometric derivative methods has the advantage of eliminating derivative steps, and consequently enhances the signal-to-noise-ratio. It has been effectively applied for resolving binary and ternary mixtures with severely overlapping spectra in complex matrices [43,44,45,46,47].

The main challenge of multicomponent analysis is the determination of more than two compounds in the same mixture without preliminary separation. The authors used in the first method MCR based on the following criteria [43,44,45,46,47],

Vector of Absorbance (Va) = α_CS_ C_CS_ + α_Deg1_ C_Deg1_ + α_Deg2_ C_Deg2_ (1)

where α is the molar absorptivity and C is the concentration.

We will discuss the determination of CS as an example and the same criteria applies for both Deg1 and Deg2.

After division over the 1st divisor (Deg1) with certain concentration (excluding zero), the result will be:

Va = α_CS_ C_CS_/α_Deg1_ + C_Deg1_ + α_Deg2_ C_Deg2_/α_Deg1_ (2)

As the mean centering of a constant equals to zero, the following is the result after MC excluding C_Deg1_

MC [Va] = MC [α_CS_ C_CS_/α_Deg1_] + MC [α_Deg2_ C_Deg2_/α_Deg1_] (3)

Finally after dividing over MC [α_Deg2_/α_Deg1_], the result will be

MC [Va]/MC [α_Deg2_/α_Deg1_] = MC [α_CS_ C_CS_/α_Deg1_]/MC [α_Deg2_/α_Deg1_] + C_Deg2_ (4)

As mean centering of a constant equals to zero, the result after MC will exclude C_Deg2_ leaving concentration of CS as the only variable against the selected amplitude.

Regarding the method optimization, the selection of the divisor concentration will have great impact on the resolution and the determination of CS, Deg1 and Deg2. Spectral features should be evident and zero absorbance should be prevented within the measured range. To achieve minimum noise and better selectivity, several divisors were tested. Good results were obtained upon using 30 μg mL^−1^ of Deg1 as the divisor to obtain the first ratio spectra of CS and Deg2 and 30 μg mL^−1^ of CS as the divisor to obtain the first ratio spectra of Deg1.

**Figure 3:**
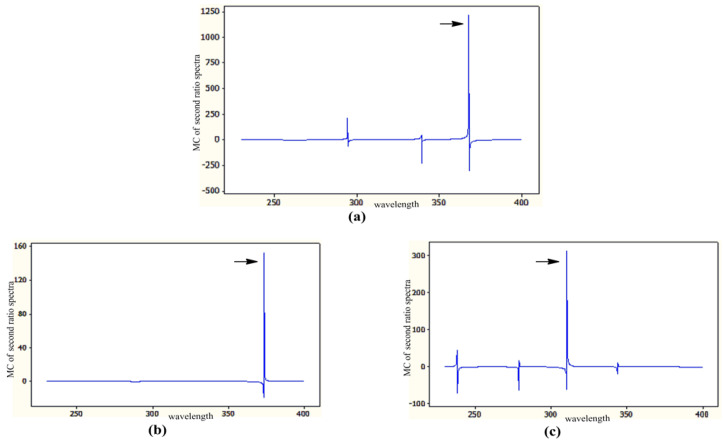
Mean center of second ratio spectra obtained for (**a**) CS at 367.8 nm, (**b**) Deg1 at 373.8 nm and (**c**) Deg2 at 310.6 nm.

Eventually, the vertical distance from peak to peak at 367.8, 373.8, and 310.6 nm was selected for CS, Deg1 and Deg2, respectively, as shown in Figure 3.

Regarding the method validation, the linearity of the anticipated method was assessed in the concentration ranges of 2–40, 5–40 and 10–100 µg mL^−1^ for CS, Deg1 and Deg2, respectively, with good regression coefficient (close to unity), LOD and LOQ (computed as, LOD = 3.3 (ơ/S) and LOQ = 10 (ơ/S), where “ơ” represents standard deviation of the intercept and “S” is the slope of the calibration line) as shown in Table 1. To assess the accuracy, intraday and the interday precision, the procedure was repeated three times (within the same day and on three successive days).

For assessing the selectivity, different ratios of CS, Deg1 and Deg2 were investigated as presented in Table 2. The results reveal that the MCR method is selective for determination of this ternary mixture without interference. Moreover, selectivity of the MCR method was ensured by selectively and successfully determining the drug in the pharmaceutical dosage form without any interference from the excipients. Good recoveries as shown in Table 2 ensured the method selectivity and suitability for different ratios and different concentrations of CS, Deg1 and Deg2.

### 3.2. Chemometric PCR and PLS Methods

Principal component regression (PCR) and partial least square (PLS-2) methods were applied. Chemometric analysis has long been used by many researchers as a multivariate advanced statistical method for the design of optimum procedures [46,47,48,49,50,51,52,53,54,55,56,57,58]. PLS is one of the chemometric multivariate calibration methods. It is based on factor analysis where PLS-1 and PLS-2 types have been termed with the known advantage that PLS-2 computes the number of factors for all studied analytes simultaneously. In PCR, the spectra decomposition is based on the maximum variance among spectral records while information about concentrations is not used, whereas for PLS both spectral and concentration data are used in modeling. Thus, PLS is allied to PCR as the spectral decomposition is performed, but this decomposition step is implemented differently.

Predictions were made of 25 mixtures of the three studied compounds using the experimental design [48], 18 as a calibration set and 7 as a validation set (Table 3). Resolving the overlap between the three studied compounds was successfully employed by using PCR and PLS-2 methods after scanning in the range of 230–400 nm with 0.2 nm intervals (851 data points). A data matrix of 25 rows and 851 columns was achieved. Then, PCR and PLS-2 were used to analyze the data after mean centering as a preprocessing step [59] and leave-one-out as a cross-validation method [60].

**Figure 4:**
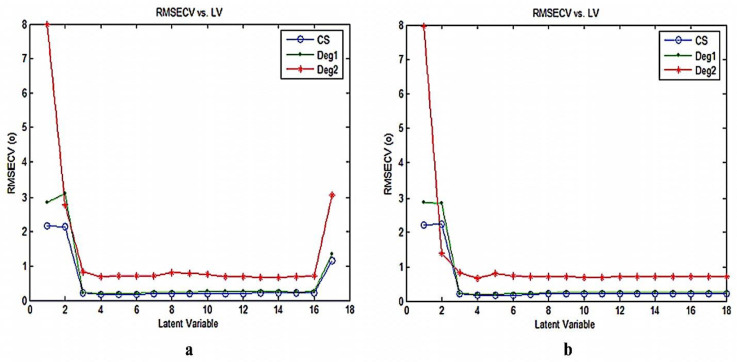
Root mean squares of error of calibration (RMSEC) plot of the cross validation results of the training set as a function of the number of principle components used to construct (**a**) the PCR and (**b**) PLS-2 calibration using zero order spectra of CS, Deg1 and Deg2.

Optimum number of factors for the underlying methods is considered to be an imperative step for accomplishing appropriate quantitation, since if the number of factors retained was too large, more noise will be further added to the data. Whereas, if the number of factors was too small, expressive data necessary for the calibration might be discarded. The choice of the finest number of substantial latent variables was designated according to Haaland and Thomas criteria [50]. Root mean squares of error of cross validation (RMSECV) values of distinctive established models were compared and in our study, the optimum number of latent variables LVs described by the developed models was found to be 4 factors for all components as shown in (Figure 4). This model was selected so that the RMSECV was not notably greater than the RMSECV from the model with an additional factor. Besides, as the difference between the lowest RMSECV and other RMSECV values decreased, the probability that a further factor was significant become smaller.

**Figure 5:**
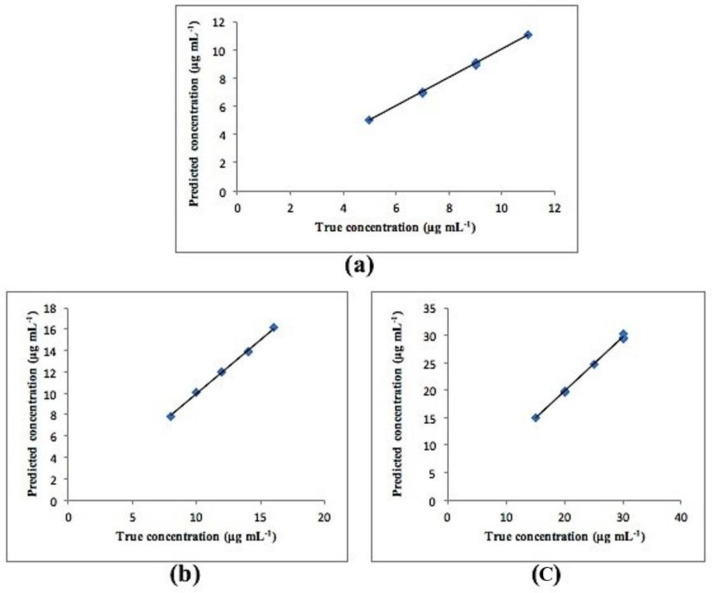
Calibration curve between true and found concentrations for (**a**) CS, (**b**) Deg1 and (**c**) Deg2 for PCR model.

**Figure 6:**
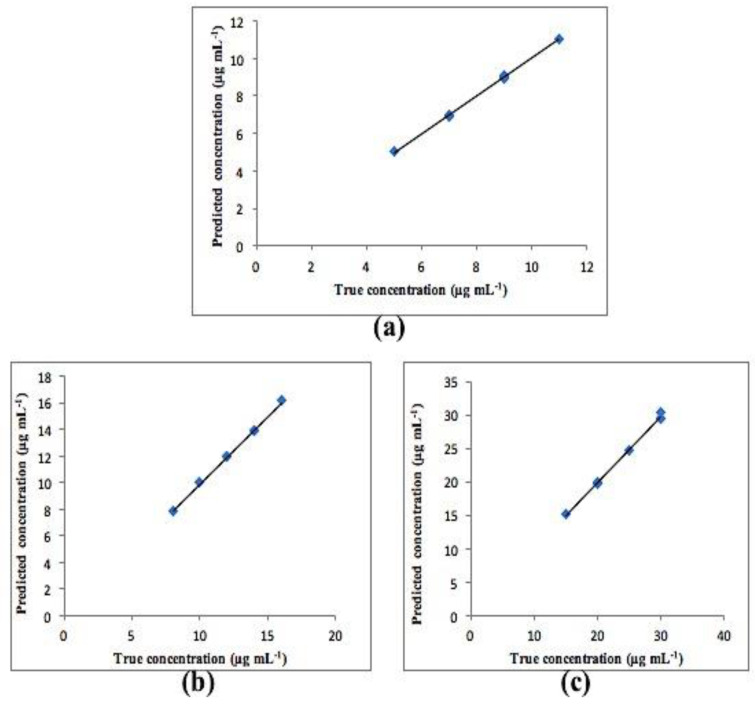
Calibration curve between true and found concentrations for (**a**) CS, (**b**) Deg1 and (**c**) Deg2 for PLS-2 model.

**Figure 7:**
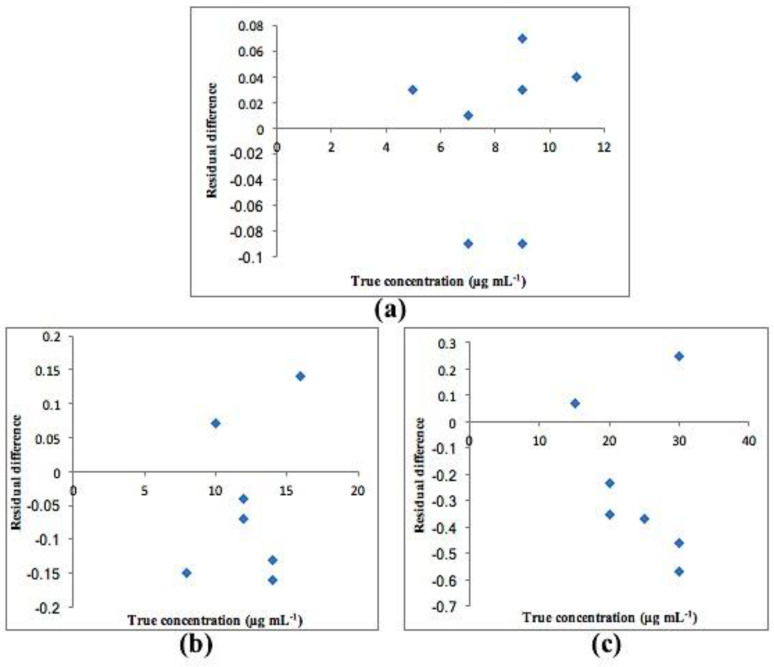
Residual plot of residual difference between true and found concentrations for (**a**) CS, (**b**) Deg1 and (**c**) Deg2 for PCR model.

**Figure 8:**
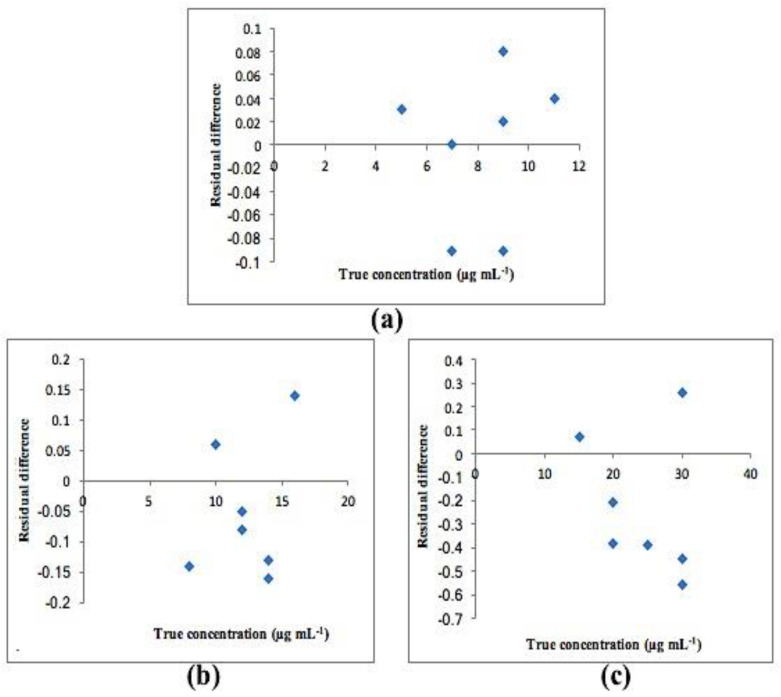
Residual plot of residual difference between true and found concentrations for (**a**) CS, (**b**) Deg1 and (**c**) Deg2 for PLS-2 model.

Calibration graphs were created by plotting the predicted concentrations for each compound versus their actual concentrations. The statistical parameters of both PCR and PLS-2 models are shown in Table 4. All plots exhibited an adequate linear relationship, a slope close to one and an intercept close to zero as shown in Figure 5 for PCR and Figure 6 for PLS-2. Residual plots were also constructed and are presented in Figure 7 and Figure 8 for PCR and PLS-2, respectively. The external validation recoveries, mean recoveries and root mean square error of prediction (RMSEP) are indicated in Table 5. The RMSEP was calculated rather than SD to assess the predictive ability of the adopted model, indicate the spread of concentration errors and also indicate precision and accuracy.

The results obtained by applying the developed chemometric methods revealed decreased RMSEP, and outstanding recoveries for all the analytes. Consequently, PLS-2 and PCR models can be successfully used for determination of CS and its degradates.

### 3.3. Application on Epicrom Eye Drops

The adopted methods had been successfully applied for the determination of CS and its degradates in Epicrom eye drops and the validity of these methods was further assessed by applying the standard addition technique as shown in Table 6.

## 4. Conclusions

The optimized analytical methods were shown to be suitable for resolving mixtures of CS and its alkaline degradates with severely overlapped spectra with good selectivity, accuracy and precision. The authors emphasize the importance of the stability-indicating strategy for the investigation of pharmaceutical products according to the International Conference of Harmonization (ICH) guidelines.

## Figures and Tables

**Table 1:** Results of the validation parameters of the MCR method.

Parameters	Method		
	CS	Deg1	Deg2
*λ* nm	367.8	373.8	310.6
Concentration range (µg mL^−1^)	2.00–40.00	5.00–40.00	10.00–100.00
Linearity			
Slope	92.022	12.053	11.203
Intercept	10.890	−35.255	−36.903
Correlation coefficient (*r*)	0.9999	0.9995	0.9999
Accuracy (Mean ± SD)	99.91 ± 1.33	100.28 ± 1.44	100.61 ± 1.55
Selectivity (Mean ± SD)	100.18 ± 1.29	100.79 ± 1.62	100.54 ± 1.59
Precision (% RSD)			
Repeatability ^a^	0.56%	0.94%	0.99%
Intermediate precision ^b^	0.92%	1.54%	1.85%
LOD ^c^ (µg mL^−1^)	0.22	0.37	0.95
LOQ ^c^ (µg mL^−1^)	0.67	1.13	2.89

^a^ Intraday (*n* = 3), average of three different concentrations repeated three times within the day. ^b^ Interday (*n* = 3), average of three different concentrations repeated three times on three successive days. ^c^ Limit of detection and limit of quantitation.

**Table 2:** Determination of CS, Deg1 and Deg2 in laboratory prepared mixtures by MCR method.

Mixture no.	Claimed Taken (µg mL^−1^)			MCR Method		
	CS	Deg1	Deg2	CS Recovery % *	Deg1 Recovery% *	Deg2 Recovery % *
1	13	10	30	100.46	100.70	99.03
2	7	12	10	100.14	101.58	102.10
3	9	12	20	99.22	98.03	98.65
4	11	16	25	102.18	101.56	101.20
5	9	10	15	98.88	102.10	101.73
Mean ± SD				100.18 ± 1.29	100.79 ± 1.62	100.54 ± 1.59

* Mean of three determinations.

**Table 3:** Concentrations of different mixtures of CS, Deg1 and Deg2 used in the calibration and validation sets in principal component regression (PCR) and partial least square (PLS-2) methods.

Mixture no.	CS	Deg1	Deg2
1 *	9	12	20
2	9	8	10
3	5	8	30
4	5	16	10
5	13	10	30
6	7	16	20
7	13	12	15
8	9	10	15
9	7	10	25
10 *	7	14	30
11	11	16	25
12	13	14	20
13 *	11	12	30
14 *	9	16	30
15	13	16	10
16	13	8	25
17	5	14	10
18	11	8	20
19	5	12	25
20 *	9	14	25
21	11	14	15
22	11	10	10
23 *	7	8	15
24 *	5	10	20
25	7	12	10

* Validation set.

**Table 4:** Statistical parameters for simultaneous determination of CS, Deg1 and Deg2 using PCR and PLS-2.

Parameters	PCR			PLS-2		
	CS	Deg1	Deg2	CS	Deg1	Deg2
Conc. Range (µg mL^−1^)	5.00–13.00	8.00–16.00	10.00–30.00	5.00–13.00	8.00–16.00	10.00–30.00
No. of Factors	4	4	4	4	4	4
RMSEC ^a^	0.13005	0.13643	0.47471	0.13077	0.13798	0.46723
RMSEP ^b^	0.09055	0.17889	0.55263	0.09220	0.17720	0.55654
RMSECV ^c^	0.16976	0.19893	0.68146	0.171	0.19925	0.67982
Arithmetic mean (Conc. Range)	9.00	12.00	20.00	9.00	12.00	20.00
(%RSD) for RMSEC ^d^	0.01445	0.01136	0.02373	0.01453	0.01149	0.02336
(%RSD) for RMSEP ^e^	0.01006	0.01490	0.02763	0.01024	0.01476	0.02782
(%RSD) for RMSECV ^f^	0.01886	0.01657	0.03407	0.019	0.01660	0.03399
Intercept ^g^	−0.0428	−0.2005	0.0794	−0.0483	−0.1993	0.0574
Slope ^d^	1.0053	1.0124	0.9870	1.0058	1.0120	0.9879
Correlation Coefficient (r ^d^)	0.9995	0.9992	0.9989	0.9994	0.9992	0.9988

^a^ Root mean square error of calibration. ^b^ Root mean square error of prediction. ^c^ Root mean square error of cross-validation. ^d^ %RSD for RMSEV = RMSEV/the arithmetic mean. ^e^ %RSD for RMSEP = RMSEP/the arithmetic mean. ^f^ %RSD for RMSECV = RMSECV/the arithmetic mean. ^g^ Data of the straight line plotted between predicted concentrations versus actual concentrations.

**Table 5:** Percentage recoveries of CS, Deg1 and Deg2 in the validation set using PCR and PLS-2.

Mix. No.	PCR			PLS-2		
	Found%			Found%		
	CS	Deg1	Deg2	CS	Deg1	Deg2
1	100.33	99.67	98.25	100.22	99.58	98.10
10	98.71	99.07	100.83	98.71	99.07	100.87
13	100.36	99.42	98.10	100.36	99.33	98.13
14	100.78	100.88	98.47	100.89	100.88	98.50
20	99.00	98.86	98.52	99.00	98.86	98.44
23	100.14	98.13	100.47	100.00	98.25	100.47
24	100.60	100.70	98.85	100.60	100.60	98.95
Mean	99.99	99.53	99.07	99.97	99.51	99.07
RMSEP *	0.09055	0.17889	0.55263	0.09220	0.17720	0.55654

* Root mean square error of prediction.

**Table 6:** Determination of CS in its pharmaceutical formulation by the proposed methods and application of standard addition technique.

Pharmaceutical Formulation	MCR			PCR			PLS		
Epicrom Eye Drops Labeled to Contain 40 mg of CS Per 1 mL	Found * % ± SD	**Added (µg mL^−1^)**	Recovery * %	Found * % SD	**Added (µg mL^−1^)**	Recovery * %	Found * % ± SD	**Added (µg mL^−1^)**	Recovery * %
	102.96 ± 1.22	3.00	98.67	102.40 ± 0.83	1	99.00	101.75 ± 0.69	1	101.00
		5.00	101.80		2	101.50		2	99.50
		10.00	100.90		3	101.67		3	101.33

* Mean of three determinations.
